# Companion Animals Are Spillover Hosts of the Multidrug-Resistant Human Extraintestinal *Escherichia coli* Pandemic Clones ST131 and ST1193

**DOI:** 10.3389/fmicb.2020.01968

**Published:** 2020-09-02

**Authors:** Amanda K. Kidsley, Rhys T. White, Scott A. Beatson, Sugiyono Saputra, Mark A. Schembri, David Gordon, James R. Johnson, Mark O’Dea, Joanne L. Mollinger, Sam Abraham, Darren J. Trott

**Affiliations:** ^1^Australian Centre for Antimicrobial Resistance Ecology, School of Animal and Veterinary Sciences, The University of Adelaide, Roseworthy, SA, Australia; ^2^School of Chemistry and Molecular Biosciences, Australian Infectious Disease Research Centre, The University of Queensland, Brisbane, QLD, Australia; ^3^Australian Centre for Ecogenomics, The University of Queensland, Brisbane, QLD, Australia; ^4^Minneapolis Veterans Affairs Health Care System and University of Minnesota, Minneapolis, MN, United States; ^5^VA Medical Center, University of Minnesota, Minneapolis, MN, United States; ^6^Antimicrobial Resistance and Infectious Disease Laboratory, College of Science, Health, Engineering and Education, Murdoch University, Murdoch, WA, Australia; ^7^Biosercurity Queensland, Department of Agriculture and Fisheries, Brisbane, QLD, Australia

**Keywords:** *Escherichia coli*, companion animals, ST131, genomics, virulence genes

## Abstract

*Escherichia coli* sequence types 131 (ST131) and 1193 are multidrug-resistant extraintestinal pathogens that have recently spread epidemically among humans and are occasionally isolated from companion animals. This study characterized a nationwide collection of fluoroquinolone-resistant (FQ^*R*^) *E. coli* isolates from extraintestinal infections in Australian cats and dogs. For this, 59 cat and dog FQ^*R*^ clinical *E. coli* isolates (representing 6.9% of an 855-isolate collection) underwent PCR-based phylotyping and whole-genome sequencing (WGS). Isolates from commensal-associated phylogenetic groups A (14/59, 24%) and B1 (18/59, 31%) were dominant, with ST224 (10/59, 17%), and ST744 (8/59, 14%) predominating. Less prevalent were phylogenetic groups D (12/59, 20%), with ST38 (8/59, 14%) predominating, and virulence-associated phylogenetic group B2 (7/59, 12%), with ST131 predominating (6/7, 86%) and no ST1193 isolates identified. In a WGS-based comparison of 20 cat and dog-source ST131 isolates with 188 reference human and animal ST131 isolates, the cat and dog-source isolates were phylogenetically diverse. Although cat and dog-source ST131 isolates exhibited some minor sub-clustering, most were closely related to human-source ST131 strains. Furthermore, the prevalence of ST131 as a cause of FQ^*R*^ infections in Australian companion animals was relatively constant between this study and the 5-year-earlier study of Platell et al. (2010) (9/125 isolates, 7.2%). Thus, although the high degree of clonal commonality among FQ^*R*^ clinical isolates from humans vs. companion animals suggests the possibility of bi-directional between-species transmission, the much higher reported prevalence of ST131 and ST1193 among FQ^*R*^ clinical isolates from humans as compared to companion animals suggests that companion animals are spillover hosts rather than being a primary reservoir for these lineages.

## Introduction

Extraintestinal pathogenic *E. coli* (ExPEC) strains have an enhanced ability to traverse from their usual gut environment to normally sterile extraintestinal body sites and cause disease (Johnson et al., 2016). Before the 2000s most ExPEC strains were susceptible to critically important antimicrobials (CIAs) such as fluoroquinolones (FQs) and extended-spectrum cephalosporins (ESCs) (Pitout, 2012), however, multidrug resistance is increasingly common globally (Nicolas-Chanoine et al., 2014). Resistance to FQs, ESCs, and carbapenems is of special concern due these drugs’ critical role in humans for treating life-threatening infections such as urosepsis (Matsumura et al., 2017).

Emerging FQ resistance is due mostly to the expansion and spread of sequence type (ST) ST131, a clonal lineage from *E. coli* phylogenetic group B2, first identified in 2008 on three continents (Coque et al., 2008; Lau et al., 2008; Nicolas-Chanoine et al., 2008). Genomic epidemiological analysis using Bayesian estimation predicts that FQ-resistance-conferring point mutations in chromosomal genes *gyrA* and *parC* occurred around 1987 in North America, coinciding with the first clinical use of FQs in the USA in 1986 (Ben Zakour et al., 2016; Stoesser et al., 2016). Most FQ-resistant (FQ^*R*^) ST131 strains derive from a single sub-ST clade, designated clade C (Petty et al., 2014), or *H*30R (Price et al., 2013) Clade C/*H*30R in turn exhibits a prominent sub-lineage, referred to as clade C2 (Petty et al., 2014) or *H*30Rx (Johnson et al., 2016), members of which typically exhibit ESC resistance, mediated by the CTX-M-15 extended-spectrum β-lactamase (ESBL). ST131 (and, specifically, clade C/*H*30R) has become the most prevalent CIA-resistant human ExPEC lineage globally (Dautzenberg et al., 2016), causing millions of antimicrobial-resistant (AMR) infections annually (Pitout and DeVinney, 2017).

Recently, ST1193, another lineage of FQ^*R*^ group B2 *E. coli*, has also emerged as an important multidrug-resistant (MDR) human pathogen (Johnson et al., 2019; Tchesnokova et al., 2019). ST1193 was identified first in Australia as an emerging FQ^*R*^ clonal group (Platell et al., 2012), and has now been reported worldwide, including in Asia (Kim et al., 2017; Wu et al., 2017; Xia et al., 2017), North America (Tchesnokova et al., 2019), and Europe (Jorgensen et al., 2017; Valenza et al., 2019). In a recent multi-center study (United States, 2016–2017), ST1193 represented one-quarter of all FQ^*R*^ human clinical urine isolates (Tchesnokova et al., 2019).

The cause of such widespread dissemination of AMR ExPEC lineages is unclear. Host-to-host transmission, including between animals and humans, likely contributes, in view of documented colonization of multiple members of the same household with the same ExPEC strain (Johnson et al., 2016) and the occurrence of (human-associated) pandemic lineages such as ST131 and ST1193 in dogs and cats (Johnson et al., 2009c; Pomba et al., 2009; Platell et al., 2012). The first reported case of an *E. coli* ST131 infection in an animal involved a dog with a UTI in Portugal. Within-household ST131 transmission between humans and pets (particularly dogs and cats) has also been documented. Additionally, in the first description of FQ^*R*^ ST1193 as a potential pandemic clone, two isolates were from dogs. As such, companion animals may be an important reservoir of these resistant clonal lineages for acquisition by humans, and humans may be a reservoir of pathogens for their pets (Timofte et al., 2014). Notably, however, in the few relevant clinical and/or ecological studies (apart from the initial report involving ST1193), both ST131 and ST1193 have been relatively rare among companion animal ExPEC isolates (Platell et al., 2012; Pitout and DeVinney, 2017), consistent with the human reservoir being primary. Additionally, both clonal lineages have been reported recently in fecal samples from wild scavenging birds (Mukerji et al., 2019; Zurfluh et al., 2019), which suggests another potential zoonotic reservoir and/or transmission pathway.

Relevant to the human-companion animal interface, previous studies have documented the emergence of FQ^*R*^ ST131 in Australian companion animals (Platell et al., 2010, 2011a; Guo et al., 2015), as both an extraintestinal pathogen and a gastrointestinal colonizer of hospitalized dogs. Whereas Platell et al. and Guo et al. investigated companion animal-source FQ^*R*^ *E. coli* from only one region in Australia, Saputra et al. (2017) later surveyed companion animal-source *E. coli* on an Australia-wide level; they found that 9.3% of 514 dog isolates and 5% of 341 cat isolates were FQ^*R*^. Based on these findings, the present study aimed to determine the prevalence of ST131 and ST1193 among both FQ^*R*^ (both ST131 and ST1193) and FQ-susceptible (FQ^*S*^) (ST131 only) members of this Australia-wide *E. coli* isolate collection. An additional aim was to compare any identified companion animal ST131 isolates with members of a multi-national collection of human and animal ST131 isolates using whole-genome sequencing (WGS).

## Materials and Methods

### Study Isolates

This study utilized three different sources of isolates. First, 855 clinical *E. coli* isolates from dogs and cats (January 2013–January 2014), accompanied by their antimicrobial susceptibility data, were obtained from the above-mentioned nation-wide survey of antimicrobial resistance in bacterial pathogens from Australian animals (Saputra et al., 2017). The isolates had been collected from 22 government, private, and university veterinary diagnostic laboratories between January 2013 and January 2014, accompanied by a brief clinical history and laboratory submission report, without client details. In all cases, the referring diagnostic microbiologist considered the isolate significant. Of the isolates, 59 (6.9%) were FQ^*R*^ (48 dog, 11 cat) and 796 (93%) were FQ^*S*^: 445 dog, 351 cat) (Table 1). Isolates were classified as multidrug-resistant (MDR) if they showed non-susceptibility to at least one antimicrobial agent in three or more antimicrobial classes. WGS was done on these 59 FQ^*R*^ cat and dog-source *E. coli* isolates as described in section “WGS of the 59 Cat and Dog-Source FQR *E. coli* Isolates” below.

**TABLE 1:** Isolates used in this study.

**Study**	**Year**	**Country**	**FQ status**	**No. isolates**	**No. of these ST131**	**Total no. ST131 isolates**
Present Study	Jan 2013–Jan 2014	Australia	FQ^R^	59	6	11
			FQ^S^	796	5	
Platell et al., 2010	2007–2009	Australia	FQ^R^	9	9	9
Ben Zakour et al., 2016	1967–2011	Various	FQ^R^	62	62	184
			FQ^S^	38	38	
			Unknown	84	84	
Reference Strains	2005–2008	United States, United Kingdom	FQ^R^	3	3	4
	Unknown	Japan	Unknown	1	1	

Second, we established an Australian cat and dog-source ST131 collection (*n* = 20). This collection included 11 ST131 isolates [six FQ^*R*^ (4 dog, 2 cat), five FQ^*S*^ (all dog)] identified here within the above-mentioned nation-wide collection of Saputra et al. (2017). It also included nine FQ^*R*^ ST131 isolates (2007–2009; 8 dog, 1 cat) from a previous study of ST131 prevalence among human and companion animal FQ^*R*^ *E. coli* from eastern Australia (Platell et al., 2010, 2011a). A more detailed WGS and phylogenetic analysis were done on these 20 Australian cat and dog-source ST131 isolates, as described in section “WGS of the 20 Australian Cat and Dog-Source ST131 Isolates” below.

Third, the above 20 Australian cat and dog-source ST131 genomes were placed into a broader context by comparing them with 188 published diverse-source ST131 genomes (Ben Zakour et al., 2016), including the complete genomes of four established ST131 reference strains: EC958 (Totsika et al., 2011) (GenBank: HG941718), SE15 (GenBank: AP009378), JJ1886 (GenBank: CP006784), and JJ1887 (GenBank: CP014316). The final multi-national dataset included the genomes of 208 ST131 isolates, as collected between 1967 and 2014 in nine countries: The United States (*n* = 83); The United Kingdom (*n* = 27); Australia (*n* = 44); Spain (*n* = 20); Canada (*n* = 19); New Zealand (*n* = 6); India (*n* = 5); Portugal (*n* = 1); Japan (*n* = 1); and Korea (*n* = 1). In addition to the 20 Australian cat and dog-source ST131 study isolates, the multi-national ST131 genome dataset included isolates from cats (*n* = 3), dogs (*n* = 3), avian species (*n* = 7), a dolphin (*n* = 1), and a primate (*n* = 1). Single-locus variants of ST131 were counted as ST131. Isolate names and available metadata are summarized in Supplementary Table S1.

### PCR-Based Phylogenetic Grouping and ST131 Status

*E. coli* phylogenetic group was determined for the present 855 clinical *E. coli* study isolates from Australian dogs and cats (59 FQ^*R*^, 796 FQ^*S*^) by using the revised Clermont multiplex PCR assay (Clermont et al., 2013). This identified 594 isolates (7 FQ^*R*^, 587 FQ^*S*^) as belonging to group B2. The B2 isolates were then screened for ST131-specific single-nucleotide polymorphisms (SNPs) in *mdh* and *gyrB* (Johnson et al., 2009b), which identified six (86% of 7) FQ^*R*^ and five (0.9% of 587) FQ^*S*^ B2 isolates as ST131.

### WGS of the 59 Cat and Dog-Source FQ^R^ *E. coli* Isolates

DNA extraction and WGS was done on the present 59 cat and dog-source FQ^*R*^ *E. coli* study isolates (48 dog, 11 cat) using the Illumina Next Seq platform as described (Abraham et al., 2018). The resulting FASTQ files were trimmed using CLC Genomics Workbench (QIAGEN Version 12), with a quality limit of 0.01, and reads with an ambiguous base were trimmed (ambiguous limit = 1). *De novo* assembly for each isolate was also performed using the CLC Genomics Workbench, using the default settings (Rahimi et al., 2018). WGS analysis was undertaken using web-based tools available at the Centre for Genome Epidemiology (CGE) and custom BLAST databases implemented in the CLC Genomics Workbench (see Supplementary Material B).

Isolates were classified provisionally as ExPEC if they contained ≥ 2 of 5 hallmark ExPEC-associated VGs, i.e., *papA* and/or *papC*, *sfa/focDE*, *afa/draBC*, *kpsM II*, and *iutA* (Johnson et al., 2003), and as UPEC if they contained ≥ 3 of 4 hallmark UPEC-associated VGs (Virulance Genes), i.e., *chuA, fyuA, vat, and yfcV* (Spurbeck et al., 2012). Additionally, isolates classified as ST131 were analyzed to determine the presence of 12 prototypic ST131-associated VGs, i.e., *iha*, *fimH*, *sat*, *fyuA*/*irp2*, *iutA*/*iucD*, *kpsM II*, *usp*, *traT*, *ompT*, and *malX* (Riley, 2014).

### ST131 Maximum-Likelihood Analysis and Phylogenetic Tree Construction

#### WGS of the 20 Australian Cat and Dog-Source ST131 Isolates

For WGS of the 20 Australian cat and dog-source ST131 isolates, genomic DNA was extracted from overnight LB broth cultures using MoBio UltraClean Microbial DNA isolation kit (QIAGEN), per the manufacturer’s instructions. Sequencing was done using the Illumina NextSeq 500 platform (San Diego, CA, United States) at the Australian Centre for Ecogenomics^1^. For this, NextSeq DNA libraries were prepared using the Nextera XT Library Preparation Kit (Illumina) with the Nextera XT Index Kit (Illumina) and were sequenced using a NextSeq 500 2 × 150 bp High-Output v2 kit (Illumina). WGS generated a median of 3.65 million reads per sample (IQR: 2.87–4.30 million reads; range: 2.33–5.11 million reads) (Supplementary Table S2). Sequence read data have been submitted to the National Center for Biotechnology Information (NCBI) Sequence Read Archive (SRA) under BioProject PRJNA627752.

#### Multi-Continental ST131 Sequence Data

The publicly available sequence read data, which had passed previously defined quality controls (Ben Zakour et al., 2016), were downloaded from the NCBI SRA using the “prefetch” and “FASTQ-dump” tools within the SRA Toolkit v2.9.0-mac64^2^ (Supplementary Table S3). The methods used for quality control, MLST and *de novo* assembly for this dataset are available in Supplementary Material B.

#### High-Resolution Phylogenetic Reconstruction of ST131

Strain EC958 was used as the template reference genome for generating a SNP-based phylogeny for 208 multi-national ST131 isolate genomes, including the 20 newly sequenced cat and dog-source ST131 isolates from Australia and the 188 published diverse-source genomes (Price et al., 2013; Petty et al., 2014) (Supplementary Table S4). For this, the coordinates of prophage and genomic island elements in the complete chromosome of EC958 were masked (Supplementary Table S5) using the bedtools (Quinlan and Hall, 2010) v2.27.1 “maskfasta” function. The trimmed paired-end reads from the 208 ST131 isolates were mapped onto the EC958-masked chromosome using the Bowtie 2 v2.3.4.2 read aligner within the Nesoni v0.132 pipeline under default settings^3^. Pseudo-genomes were created for each strain by integrating strain-specific SNPs into the backbone of EC958, which were then aligned to generate a multiple-genome alignment (Petty et al., 2014).

All polymorphic substitution sites were concatenated into a multi-FASTA alignment that was filtered for recombination by using the Gubbins algorithm (default settings, “raxml mode” with the General Time Reversible (GTR) GAMMA correction). Removal of putatively recombined regions left 7,527 total non-recombinant core-genome SNPs. The recombination-filtered SNP alignment was imported into jModelTest v2.1.10 (Guindon and Gascuel, 2003; Darriba et al., 2012) and 12 candidate models were tested from three substitution schemes, with the base tree for likelihood calculations optimized for ML and parameters “+ F,” “+ I,” “+ G (nCat = 4).” The GTR nucleotide substitution model with GAMMA distribution was determined as the best-fit evolutionary model based on the lowest Akaike and Bayesian Information Criterion (AIC and BIC) scores.

The evolutionary history was inferred by importing this alignment into RAxML (Stamatakis, 2014) v8.2.10 (GTR-GAMMA correction) and using the ML method thorough optimization of the 20 distinct randomized Maximum Parsimony trees, before adding 1,000 bootstrap replicates. FigTree visualized the ML phylogenetic tree outputs and exported the trees in NEXUS format. NEXUS trees were visualized in EvolView v2 (Zhang et al., 2012; He et al., 2016) for final output generation. The recombination-filtered SNP alignment also was converted into a pairwise SNP distance matrix using snp-dists v0.2^4^.

## Results

### Phylogenetic Grouping of FQ^*R*^ Isolates

The present study’s 59 Australian cat and dog-source FQ^*R*^ *E. coli* isolates were distributed broadly by phylogenetic group (in order of descending prevalence) as follows: B1 (31%), A (24%), D (20%), B2 (12%), F (10%), and C (3%). Nearly all isolates qualified as MDR (56/59, 95%), and MDR isolates exhibited a similar phylogenetic group distribution as did the total population of 59 isolates (not shown).

### Whole Genome Sequenced-Based Analyses

STs, ARGs, and VGs identified for each isolate by *in silico* analysis are shown in Supplementary Table S6.

#### *In silico* MLST

**FIGURE 1:**
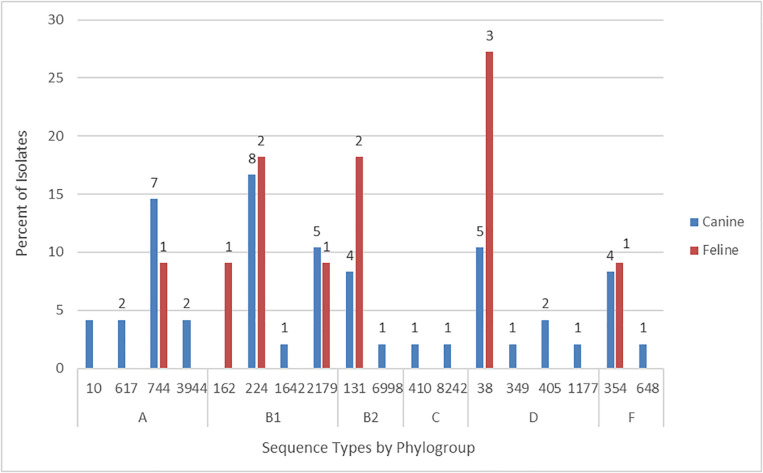
Sequence type frequency among 59 fluoroquinolone-resistant cat and dog clinical *Escherichia coli* isolates that underwent whole genome sequencing.

According to *in silico* MLST the 59 Australian cat and dog-source FQ^*R*^ isolates represented 18 STs, including one novel ST (ST8242; phylogroup C). Ten STs accounted for ≥ 2 isolates each, the other eight (including ST8242) for a single isolate each. The five most prevalent STs – ST38 (eight isolates), ST131 [six isolates (these strains had already been identified as ST131 by SNP PCR)], ST224 (10 isolates), ST744 (eight isolates), and ST2179 (six isolates) – accounted for 64% of the 59 FQ^*R*^ isolates (Figure 1). Six STs occurred among both cat and dog-source isolates (ST38, ST131, ST224, ST354, ST744, and ST2179). None of the isolates represented other major STs associated with FQ^*R*^ human UTI isolates, e.g., ST1193.

The six phylogroup F isolates from STs 354 (5 isolates) and 648 (1 isolate) were characterized previously (Vangchhia et al., 2016). Consequently, they did not undergo further genomic analysis here and are not included in the following sections, which are limited to the remaining 53 sequenced FQ^*R*^ isolates.

#### ARGs

The 53 further-analyzed FQ^*R*^ isolates contained two PMQR genes, including *aac(6’)-lb-cr* (25%) and *qnrS1* (2%). The most common CTX-M type ESBL genes were *bla*_*CTX–M–*__15_ (25%) and *bla*_*CTX–M–*__14_ (6%). The AmpC β-lactamase gene *bla*_*CMY–*__2_ was identified in 12 isolates (23%) and *bla*_*OXA–*__1_ in 13 isolates (25%). The ESBL genes *bla*_*CTX–M–*__15_, *bla*_*CTX–M–*__14_ were both identified in isolates belonging to ST38 and ST131. Isolates containing *bla*_*CTX–M–*__15_ were also assigned to ST405, ST410, and ST627, while *bla*_CTX–M–__14_-containing isolates also belonged to ST224. The AmpC β-lactamase gene *bla*_*CMY–*__2_ was identified in ST38, ST224, ST617, ST744, and ST2179, whereas *bla*_OXA–__1_ was identified in ST38, ST131, ST405, ST410, and ST617.

#### VGs

The 53 further-analyzed FQ^*R*^ isolates contained 160 distinct VGs (representing 51 operons). Of the 160 VGs, 16 (10%) occurred in all 53 isolates; these included *ompA* (outer membrane protein), *entABCDEFS* (enterobactin siderophore system), *fepACDG* (ferric enterobactin uptake), and *ecpABCDE* (*E. coli* common pilus).

The number of VGs per isolate differed by phylogroup, and within a given phylogroup differed by ST. By phylogroup, isolates from groups B2 and D contained the most VGs per isolate (medians, 80 and 88, respectively), followed by those from phylogroups B1 (median 61.5), C (median 58), and A (median 43.5) (Supplementary Table S6). By ST, isolates from ST131 (phylogroup B2) and ST38 (phylogroup D) contained the most VGs per isolate (medians 81 and 92.5, respectively), whereas those from ST744 (phylogroup A) and ST224 (group B1) contained the fewest (medians 39 and 54.5, respectively).

Nine of the 53 FQ^*R*^ isolates qualified molecularly as ExPEC (but not UPEC), one as UPEC (but not ExPEC), and one as both ExPEC and UPEC, giving 10 total ExPEC isolates (19%) and two UPEC isolates (4%). Of the 10 ExPEC isolates, nine (90%) belonged to phylogroups B2 (n = 3; all from ST131) or D (n = 6; 5 from ST38, 1 from ST349); the sole exception was from phylogroup A (ST744).

#### Serotypes, *fimH* Alleles, ARGs, and VGs in the FQ^R^ vs. FQ^S^ ST131 Isolates From Cats and Dogs in Australia

Regarding O:H serotypes and *fimH* alleles, the six FQ^*R*^ ST131 isolates exhibited two predicted combinations, O16:H5 *fimH*41 (*n* = 1) and O25:H4 *fimH*30 (*n* = 5; representing the C/*H*30R subclone). Regarding resistance characteristics, all six isolates were MDR and possessed at least two β-lactamase genes, including *bla*_*CTX–M–*__15_ (three of five C/*H*30R isolates) and *bla*_*CTX–M–*__14_ and *bla*_*CTX–M–*__27_ (one isolate each). Four isolates were MDR; two of these contained, respectively, *aac(6’)-Ib-cr* or *qnrB4*. The five FQ^*S*^ ST131 strains also exhibited two predicted serotype-*fimH* allele combinations, O16:H5 *fimH41* (*n* = 2) and O25:H4 *fimH22* (*n* = 3). Four isolates were MDR, and one each contained *aac(6’)-Ib-cr* or *qnrB4*.

Regarding virulence genotypes of FQ^R^ vs. FQ^S^ companion animal-source ST131 isolates, the six dog or cat-source FQ^R^ ST131 isolates each contained 72–94 putative VGs (median 81 VGs). Although none of the six had all 12 prototypic ST131-associated VGs, i.e., *iha*, *fimH*, *sat*, *fyuA*/*irp2*, *iutA*/*iucD*, *kpsM II*, *usp*, *traT*, *ompT*, and *malX* (Riley, 2014), three contained all 12 of these genes except *iutA* and *ompT*, while the other three additionally lacked only *usp*. Three of the six FQ^*R*^ ST131 isolates qualified as ExPEC but none as UPEC. By comparison, the five FQ^S^ dog-source ST131 isolates each contained 71-100 VGs (median 79 VGs). Like the FQ ST131 strains, none contained all 12 prototypic ST131-associated VGs, however, one isolate contained all of these except *iutA* and *ompT*, whereas the rest contained ≤ 9 of the 12 genes each. One dog-source FQ^*S*^ ST131 isolate qualified as both ExPEC and UPEC, one as ExPEC only, and one as UPEC only. Thus, FQ^*R*^ and FQ^*S*^ companion animal ST131 isolates from Australia possess similar virulence characteristics.

#### Phylogenetic Relationships Among 208 Multi-National ST131 Isolates, Including 20 From Australian Cats and Dogs and 188 From Diverse Host Species in Other Locales

**FIGURE 2:**
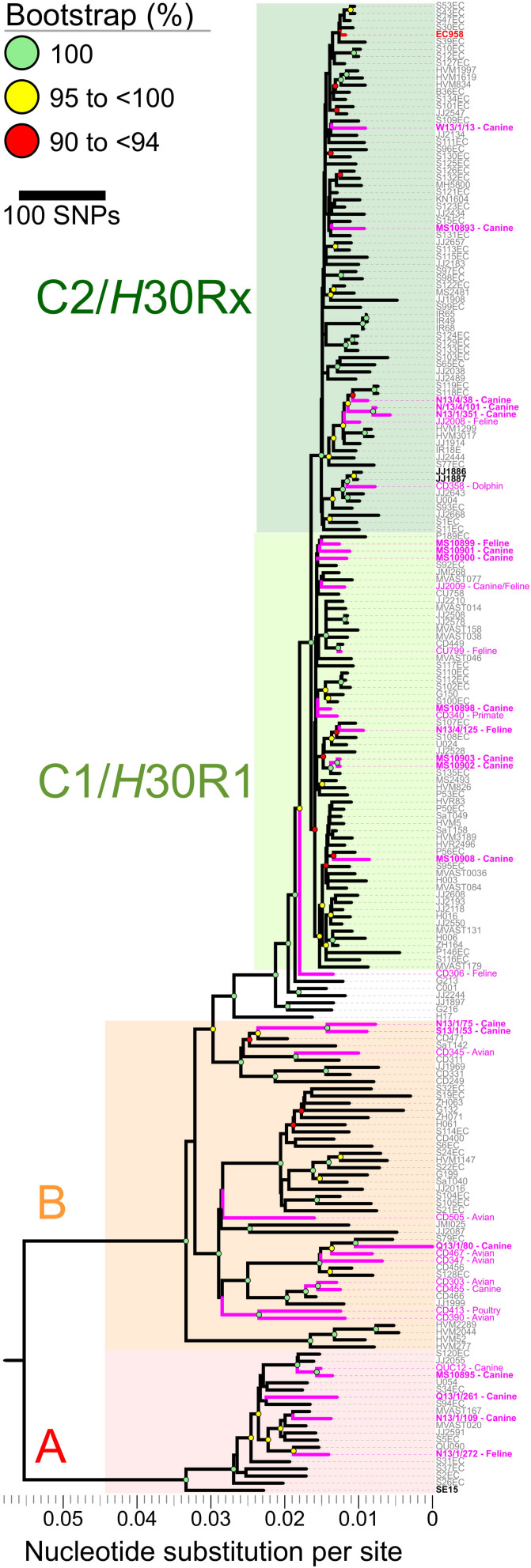
Core-genome single nucleotide polymorphisms (SNPs) phylogeny of 208 *Escherichia coli* ST131 isolates from humans, cats, dogs, and other animals. The Maximum Likelihood tree was built using 7,527 non-recombinant core-genome single nucleotide polymorphisms (SNPs), as called by mapping sequencing reads to the chromosome of EC958 (GenBank: HG941718.1) with masked genomic islands and prophage regions. Colored groupings represent three labeled clades. *H*41/A (red), *H*22/B (orange), and C1/C2/*H*30 (green). Branch lengths are drawn to scale and represent the number of nucleotide substitutions per site (scalebar) or number of SNPs (key). Pink labels represent isolates collected from animals. Boldface pink labels with an asterisk represent the 20 cat and dog-source ST131 isolates sequenced as part of this study (11 from the Saputra et al. (2017) study, and nine [indicated by the MS prefix] from the Platell et al. (2010) study).

Mapping of Illumina reads from the 208 diverse-source ST131 isolates to the EC958 ST131 reference chromosome identified 7,527 non-recombinant core-genome SNPs (Figure 2). After masking of SNPs within the 577,661 bp of identified prophage and genomic island sequence (11.3% of the EC958 genome) a ML phylogeny was inferred. The phylogeny had strong bootstrap support for its topology, which matched that of previous reports (Price et al., 2013; Ben Zakour et al., 2016), including resolution of the three major ST131 clades (A, B, and C, corresponding to the *H*41, *H*22 and *H*30 *fimH* fimbrial types). Most of the 20 Australian cat and dog-source ST131 isolates fell within Clade C/*H*30 (13/20, 65%; 8/13, 62% in C1/*H*30R1 and 5/13, 38% in C2/*H*30Rx), followed distantly by Clades A/*H*41 (4/20, 20%) and B/*H*22 (3/20, 15%) (Figure 2).

Notwithstanding the absence of an obvious animal-only clade, seven of the 20 Australian cat and dog-source ST131 isolates clustered closely within three sub-clades (Figure 2). (i) C1/*H*30R1 strains MS10902 and MS10903, which differed by only six SNPs, were dog-source urine isolates from Victoria (Platell et al., 2010). (ii) C2/*H*30Rx strains N13/4/101 and N13/1/351, which differed by only 20 SNPs, were dog urine isolates from New South Wales that clustered with strain N13/4/38 (dog trachea biopsy isolate, also from New South Wales) in another sub-clade (Saputra et al., 2017). These differed by only 42–72 SNPs from two human isolates collected in 2011 in the United Kingdom (strains S118EC and S119EC). Strain JJ2008, a cat isolate collected in the United States, formed an outgroup to this mixed human/animal sub-clade (Figure 2). (iii) The remaining two isolates, *H*22/B strains N13/1/75 and S13/1/53 (dog-source urine and ear swab isolates from New South Wales and South Australia, respectively) (Saputra et al., 2017), formed a sub-clade and differed from one another by only 96 SNPs (Figure 2).

## Discussion

This study, which sought to characterize genetically a nationwide collection of clinical FQ^*R*^ *E. coli* isolates from extraintestinal infections in Australian dogs and cats (January 2013–January 2014) – including a WGS-based phylogenetic comparison across host species and geographical regions – led to four main conclusions. First, most (58%) FQ^*R*^ dog and cat isolates represented commensal phylogenetic groups A (24%), B1 (31%), and C (3%), rather than the group B2-derived pandemic lineages (ST131 and ST1193) that predominate among human FQ^*R*^ isolates. Second, the prevalence of ST131 as a cause of FQ^*R*^ *E. coli* infections in Australian companion animals was similarly low here among comparatively recent isolates (January 2013–January 2014) (6/59, 10%) as that noted previously by Platell et al. among historical isolates (2007–2009) (9/125, 7.2%), suggesting stability over time. Third, in a SNP-based phylogeny, the ST131 isolates from companion animals and humans were intermingled throughout the tree, rather than forming host-specific clades. This, together with the comparatively low prevalence of ST131 among isolates from companion animals, supports the hypothesis that companion animals in Australia are spillover hosts for ST131. Fourth, the absence of ST1193 among the present companion animal-source FQ^*R*^ *E. coli* isolates and its rarity in other companion animal-source isolate collections suggests that the same spillover hypothesis likely applies also to this clonal group, which is emerging among humans but not in animals.

Regarding our first main conclusion, according to most studies ExPEC derive mainly from phylogroups B2, D, and F, and non-pathogenic commensal *E. coli* from phylogroups A and B1 (Johnson and Stell, 2000; Vangchhia et al., 2016). Today, resistance to FQs and ESCs among human-source clinical *E. coli* is due mainly to the group B2-derived lineages ST131 and ST1193, most members of which qualify as ExPEC (Johnson et al., 2019; Tchesnokova et al., 2019). By contrast, prior to these two clonal groups’ recent emergence and expansion, most FQ^*R*^ human-source clinical isolates belonged to groups A and B1, and typically contained fewer VGs than did FQ^*S*^ isolates (Clermont et al., 2000).

The present study confirms that this historic pattern of phylogroup distribution persists among recent dog and cat-source FQ^*R*^ clinical *E. coli* isolates from Australia. Whilst the overall prevalence of FQ resistance in the Australia-wide collection of companion animal *E. coli* is low (< 10%) by international standards (Saputra et al., 2017), the FQ^*R*^ isolates belonged predominantly to groups A and B1 and possessed fewer VGs than group B2 and D isolates, with only seven of 53 (13%) FQ^*R*^ non-ST131 isolates qualifying molecularly as ExPEC. These findings echo a comparable study conducted over 10 years ago on *E. coli* isolates from North American dogs with UTI, in which FQ^R^ differed significantly in VG content (fewer) and phylogenetic background (mostly non-group B2) from FQS isolates (more VGs, mostly B2) (Johnson et al., 2009a).

Regarding our second main conclusion, comparison of the present findings with those of the regionally based study of Platell et al. (2010) confirmed that the prevalence of ST131 as a cause of FQ^*R*^ clinical infections in Australian companion animals did not increase significantly over the 5 year between-study interval. Two recent studies documented ST131 as a cause of extraintestinal infections in companion animals in Europe, but identified different proportions of C1/*H*30R1 vs. C2/*H*30Rx among cat and dog-source isolates (Belas et al., 2018; Melo et al., 2019). However, neither included a detailed genomic comparison with human ST131 isolates. Specifically, among urine *E. coli* isolates from cats and dogs in Portugal (1999–2015), 15% (25/172) of group B2 isolates were ST131, of which seven (28%) represented C/*H*30. Three (43%) of the C/*H*30 isolates represented the C2/*H*30Rx subclade, and two of these possessed *bla*_*CTX–M–*__15_ (Belas et al., 2018). Likewise, among more recent ESC-resistant *E. coli* isolates from cats and dogs in France (2010–16), most ST131 isolates represented C/*H*30 (50/56, 89%), 32 (64%) of which represented the C2/*H*30Rx subclade, and several possessed *bla*_*CTX–M–*__27_ (Melo et al., 2019). By contrast, 36% (4/11) of the present cat and dog ST131 isolates (one of which was identified as carrying the *bla*_*CTX–M–*__27_ gene), and only 11% (1/9) of those studied by Platell et al. (2010), represented the C2/*H*30Rx subclade. Taken together, these four studies suggest that the C2/*H*30Rx clade is becoming more common as a cause of extraintestinal infection in companion animals. A larger multicenter study involving genomic comparison of more recent companion animal ST131 isolates is needed to confirm this hypothesis.

Regarding human-companion animal ST131 clonal commonality, our comparison of 20 Australian cat and dog-source ST131 genomes with 173 human-source ST131 genomes (Ben Zakour et al., 2016) confirmed that the animal-source isolates were widely distributed throughout the ST131 phylogeny, in many cases placed closely to human ST131 strains. The absence of a separate clade containing only companion animal ST131 strains suggests a spillover model for ST131 occurrence in companion animals, as hypothesized previously (Bourne et al., 2019). Indeed, some animal ST131 strains shared very high identity with human strains. For example, MS10899, an Australian cat urine isolate (2007–2009), differed from P189EC, a Spanish human fecal isolate (2011), by only 66 non-recombinant core-genome SNPs (Figure 2). Although we cannot exclude that an individual from Spain came into contact with a cat in Australia in 2005, a more plausible explanation is the existence of an as-yet undefined reservoir or transmission network of circulating ST131 strains that links Australia and Spain (and, likely, much of the rest of the world).

Regarding our third main conclusion, human-animal clonal commonality implies interspecies transmission potential (of indeterminate direction), whereas the comparative prevalence of ST131 among human vs. animal clinical isolates implicates humans rather than companion animals as the main reservoir. Combined with the fact that the relative prevalence of ST131 among clinical isolates from dogs in Australia was fairly stable between 2007 and 2009 (Platell et al., 2010) and 2013 (this study), this adds further support for the hypothesis that ST131 causes infections predominantly in humans, with cats and dogs representing occasional spillover hosts (Melo et al., 2019). It is plausible, however, that pets may carry significant numbers of ST131 strains in the gut and could facilitate interspecies transfer to humans, who may simply be more susceptible than pets to developing extraintestinal infection due to ST131. By contrast, pets may be predisposed to developing infection due to other *E. coli* STs such as ST372, which is comparatively rarer among humans (LeCuyer et al., 2018; Kidsley et al., 2020; Valat et al., 2020).

Despite the overall genetic diversity of the Australian cat and dog-source ST131 isolates, some sub-clustering was apparent within the reference strain ST131 phylogeny. Based on the geographical, temporal, and host species metadata and minimal SNP diversity, it is likely that a single epidemiological source (per cluster) gave rise to each of the three clusters of closely related companion animal isolates within ST131 clades B, C1/*H*30R1, and C2/*H*30Rx. Given the distribution of the 34 animal ST131 isolates within the SNP-based phylogeny and the spillover hypothesis, further work will be required to characterize the reservoirs and transmission pathways that facilitate the spread of ST131 beyond the human population (Platell et al., 2011b).

Regarding our fourth main conclusion, ST1193 – another emerging clonal lineage of FQ^*R*^ *E. coli* within phylogroup B2 (Johnson et al., 2019; Tchesnokova et al., 2019; Valenza et al., 2019) — accounted for none of the 53 present FQ^*R*^ cat and dog isolates, despite having accounted for two FQ^*R*^ dog clinical isolates from Australia in the earlier Platell et al. study (Platell et al., 2012). To our knowledge only two other studies have recovered ST1193 from companion animals. Maeyama et al. (2018) identified five ESC resistant ST1193 isolates among 381 clinical *E. coli* isolates from companion animals in Japan in 2016 (Maeyama et al., 2018); while Zhang et al. (2018) identified one (*n* = 24) CTX-M-positive ST1193 of clinical origin in Canada (Zhang et al., 2018). ST1193’s rarity of isolation from companion animals suggests that ST1193, like ST131, is likely is predominantly a human-source clone, for which dogs are mainly spillover hosts. Regarding possible alternate transmission pathways for ST1193, in a recent Australian study a high proportion of silver gulls carried ESC-resistant and FQ^*R*^ *E. coli* in their feces. Whilst the most commonly identified ST was ST131 (17%), a substantial proportion of isolates represented ST1193 (6%), and these were closely related to human isolates (Mukerji et al., 2019).

This study has relevant limitations. First, selection bias may have influenced the observed distribution of FQ^*R*^ STs, since the study isolates came from veterinary diagnostic laboratories, which preferentially receive specimens from complicated cases and hosts with prior antimicrobial therapy (unpublished data). Second, detailed clinical data were lacking. Third, the clinical impact of the observed *in vitro* and genotypically predicted antimicrobial resistance is unknown. The study also has strengths: these include the large sample size (*n* = 855, making it the largest such study to date), the “snapshot” 1-year study period, the broad geographical sampling across Australia, and the genomic comparison with a large multi-national collection of (predominantly human-source) reference ST131 genomes.

## Conclusion

In conclusion, we found that despite ST131 being the most prevalent ExPEC strain reported globally among humans, it occurred only at comparatively low frequency among FQ^*R*^ and FQ^*S*^ *E. coli* clinical isolates from Australian cats and dogs over a 1-year period in 2013–2014 (10%), with little change since 2007–2009 (7.2%). Phylogenomic comparisons of ST131 isolates from humans, cats, and dogs in Australia showed that despite some sub-clustering, the animal isolates were widely distributed throughout the ST131 phylogeny, often closely resembling human isolates. By contrast with ST131, ST1193, although identified first as an emerging clonal group among Australian FQ^*R*^ human and animal-source *E. coli* from 2007–2009 (Platell et al., 2012), was absent from the present FQ^*R*^ dog and cat isolate collection.

## Data Availability Statement

The datasets presented in this study can be found in online repositories. The names of the repository/repositories and accession number(s) can be found at: Illumina sequence data for all 20 isolates have been deposited to the SRA under the accession numbers SRR11608154–SRR11608173 (BioProject PRJNA627752).

## Author Contributions

AK performed the experiments and data analysis, and drafted and prepared the manuscript. RW performed the whole genome sequencing and built and analyzed the phylogenetic tree. SS performed the initial antimicrobial susceptibility testing. JM provided the additional ST131 isolates. SB, MS, MO’D, DG, JJ, SA, and DT were involved in the experimental design development and manuscript preparation. All authors contributed to the article and approved the submitted version.

## Conflict of Interest

DT has received research funding and undertaken consultancies for Bayer, Zoetis, Boehringer Ingelheim, Virbac, Luoda Pharma, Neoculi, and IRiccorgpharm. SA has received research funding from Zoetis and Neoculi. JJ has received research support from and/or has undertaken consultancies for Achaogen, Allergan, Crucell/Janssen, Melinta, Merck, Shionogi, Syntiron, and Tetraphase. The remaining authors declare that the research was conducted in the absence of any commercial or financial relationships that could be construed as a potential conflict of interest.

## Footnotes

1 http://ecogenomic.org/services

2 http://ncbi.github.io/sra-tools

3 https://github.com/Victorian-Bioinformatics-Consortium/nesoni

4 https://github.com/tseemann/snp-dists
