# Supplementary material for: Companion Animals Are Spillover Hosts of the Multidrug-Resistant Human Extraintestinal Escherichia coli Pandemic Clones ST131 and ST1193
Source: Front Microbiol. 2020 Sep 2;11:1968. doi: 10.3389/fmicb.2020.01968 (PMC7492567; doi:10.3389/fmicb.2020.01968)
Supplement: Supplementary file 3 [file Data_Sheet_3.docx]

# Supplementary materials and methods

**WGS analysis of 59 cat and dog-source FQ^R^ *E. coli* isolates**

For each isolate, assembled contigs were analyzed for sequence type (ST), antimicrobial resistance genes (ARGs), virulence genes (VGs), plasmids, *fimH* alleles, and O:H serotypes by using web-services at the Centre for Genome Epidemiology (CGE, http://www.genomicepidemiology.org/), and the ABRicate function of the Galaxy web platform (https://usegalaxy.org.au/). Isolates of unknown ST per CGE were uploaded to Enterobase for further analysis (http://enterobase.warwick.ac.uk/).

A BLAST library was constructed for *afaBC* (NCBI X76688), *draBC* (NCBI AF329316), *traT* (NCBI CP001856), *usp* (NCBI AB027193), *ompT* (NCBI AE014075), *malX* (NCBI AE014075), and *yfcV* (NCBI AE014075), and was used to search contigs using CLC Genomics Workbench. *kpsM II* and *hra* were detected by performing a local blastn search using the sequences in Supplementary Data Sheet 4.

### Additional methods supporting ST131 phylogenetic tree construction

### Quality control and MLST

DNA contamination was screened within the Illumina sequence data by using Kraken v2.0.7-beta (Wood and Salzberg, 2014) against the NCBI Reference Sequence (RefSeq) database (Sayers et al., 2009). Next the quality of the paired-end reads was assessed by using the FastQC package v0.11.8 (http://www.bioinformatics.babraham.ac.uk/projects/fastqc/) by aggregating all reports into a single report and visualizing them using MultiQC v1.7 (Ewels et al., 2016). Low-quality bases, read-pairs and Illumina adaptor sequences were removed using Trimmomatic v0.36 (Bolger et al., 2014) (settings: LEADING:10 TRAILING:10 MINLEN:50 HEADCROP:10). Quality filtering with Trimmomatic retained 99.6% of read pairs (Table S2). SRST2 v0.2.0 (Inouye et al., 2014) (default settings) was used to screen for MLST genes by querying all genomes against the *E. coli* MLST allelic profiles hosted on PubMLST (Wirth et al., 2006; Larsen et al., 2012).

### *De novo* assembly of Australian cat and dog-source ST131 draft genomes

Using the chromosome of ST131 reference strain EC958, a reference-assisted approach was taken to generate *de novo* assemblies of the 20 newly sequenced Australian cat and dog-source ST131 isolate genomes using MGAP (https://github.com/dsarov/MGAP Microbial-Genome-Assembler-Pipeline). By comparing draft genome assemblies to the chromosome of EC958, assembly statistics were generated using QUAST v4.5 (Gurevich et al., 2013). The 20 Australian draft genomes had a median total length of 5.18 Mbp (IQR: 5.05 to 5.27 Mbp; range: 5.02 Mbp to 6.39 Mbp), a median GC content of 50.7% (IQR: 50.7% to 50.8%; range: 50.3% to 50.9%), and a median N50 statistic of 229.9 Kbp (IQR: 218.6 to 246.3 Kbp; range: 52.1 to 370.4 Kbp) (Table S2).

**References**

Bolger, A.M., Lohse, M., and Usadel, B. (2014). Trimmomatic: a flexible trimmer for Illumina sequence data. *Bioinformatics* 30(15)**,** 2114-2120. doi: 10.1093/bioinformatics/btu170.

Ewels, P., Magnusson, M., Lundin, S., and Kaller, M. (2016). MultiQC: summarize analysis results for multiple tools and samples in a single report. *Bioinformatics* 32(19)**,** 3047-3048. doi: 10.1093/bioinformatics/btw354.

Gurevich, A., Saveliev, V., Vyahhi, N., and Tesler, G. (2013). QUAST: quality assessment tool for genome assemblies. *Bioinformatics* 29(8)**,** 1072-1075. doi: 10.1093/bioinformatics/btt086.

Inouye, M., Dashnow, H., Raven, L.A., Schultz, M.B., Pope, B.J., Tomita, T., et al. (2014). SRST2: Rapid genomic surveillance for public health and hospital microbiology labs. *Genome Med.* 6(11)**,** 90. doi: 10.1186/s13073-014-0090-6.

Larsen, M.V., Cosentino, S., Rasmussen, S., Friis, C., Hasman, H., Marvig, R.L., et al. (2012). Multilocus sequence typing of total-genome-sequenced bacteria. *J. Clin. Microbiol.* 50(4)**,** 1355-1361. doi: 10.1128/jcm.06094-11.

Sayers, E.W., Barrett, T., Benson, D.A., Bryant, S.H., Canese, K., Chetvernin, V., et al. (2009). Database resources of the National Center for Biotechnology Information (vol 37, pg D5, 2008). *Nucleic Acids Res*. 37(9)**,** 3124-3124. doi: 10.1093/nar/gkp382.

Wirth, T., Falush, D., Lan, R.T., Colles, F., Mensa, P., Wieler, L.H., et al. (2006). Sex and virulence in Escherichia coli: an evolutionary perspective. *Molecular Microbiol*. 60(5)**,** 1136-1151. doi: 10.1111/j.1365-2958.2006.05172.x.

Wood, D.E., and Salzberg, S.L. (2014). Kraken: ultrafast metagenomic sequence classification using exact alignments. *Genome Biol.* 15(3)**,** R46. doi: 10.1186/gb-2014-15-3-r46.
